# Complete Genome Sequence of a *Bohle iridovirus* Isolate from Ornate Burrowing Frogs (*Limnodynastes ornatus*) in Australia

**DOI:** 10.1128/genomeA.00632-16

**Published:** 2016-08-18

**Authors:** Paul M. Hick, Kuttichantran Subramaniam, Patrick Thompson, Richard J. Whittington, Thomas B. Waltzek

**Affiliations:** a OIE Reference Laboratory for Epizootic Hematopoietic Necrosis Virus and Ranavirus Infection of Amphibians, Faculty of Veterinary Science, School of Life and Environment Sciences, The University of Sydney, Sydney, Australia; b Department of Infectious Diseases and Pathology, College of Veterinary Medicine, University of Florida, Gainesville, Florida, USA

## Abstract

*Bohle iridovirus* (BIV) is a species within the genus *Ranavirus*, family *Iridoviridae*, first isolated from the ornate burrowing frog *Limnodynastes ornatus* in Australia. The BIV genome confirms it is closely related to isolates from boreal toad *Anaxyrus boreas* and leaf-tailed gecko *Uroplatus fimbriatus* within the United States and Germany, respectively.

## GENOME ANNOUNCEMENT

*Bohle iridovirus* (BIV) was first isolated from ornate burrowing frogs that died at the time of metamorphosis (1). Although rarely recognized in natural disease, experimental studies indicate that BIV is a potential pathogen of fish (2, 3), amphibians (4), and reptiles (5). Infection of amphibians with viruses of the genus *Ranavirus*, including BIV, is a disease listed by the World Organisation for Animal Health because of the potential to cause ecologically and economically important epizootics (6). There is a risk of these viruses spreading to new locations and naive hosts through trade in susceptible ornamental and food species (7). Until recently, BIV was isolated only from diseased amphibians in northern Australia (1, 8). However, strains of BIV have now been identified in diseased boreal toads in a mixed-species captive population in Iowa, USA (9), and a diseased leaf-tailed gecko as part of a mixed collection of reptiles and amphibians in Germany (10, 11).

A freeze-dried stock of third passage cell culture supernatant containing the isolate BIV-ME 93/35 isolated from burrowing frog was amplified using BF-2 cells maintained with Dulbecco’s modified Eagle’s medium with 5% fetal bovine serum at 22°C. Inoculation of BF-2 cells at a high multiplicity of infection provided fifth passage material harvested after 48 h when cytopathic effect was extensive. The cell culture supernatant was clarified at 3,000 × *g* for 20 min, and total nucleic acids were purified from the supernatant using a High Pure viral nucleic acid kit (Roche). A library was prepared using the Nextera XT DNA kit, and sequencing was performed using a v3 chemistry 600-cycle kit on a MiSeq platform (Illumina). *De novo* assembly of 6,274,206 paired-end reads was performed in SPAdes (12), producing a contiguous consensus sequence of 103,531 bp, with a G+C content of 55.2%. The quality of the BIV-ME 93/35 assembly was verified by mapping the reads back to the consensus sequence in Bowtie 2 (13) and visually inspecting the alignment in Tablet (14). A total of 5,198,435 reads (82.85%) aligned at an average coverage of 10,744 reads/nucleotide. The size of the BIV-ME 93/35 genome was comparable to that of the 103,681-bp German Gecko ranavirus (GGRV; GenBank accession no. KP266742) and smaller than that of the 105,903-bp *Frog virus 3* (FV3; GenBank accession no. NC_005946).

The genome of BIV-ME 93/35 was annotated using GATU (15), with FV3 genome as the reference. Additional putative open reading frames (ORFs) were identified using GeneMarkS (16). One hundred putative ORFs were identified, and a phylogenetic analysis based on the concatenated nucleotide sequences of the 26 *Iridoviridae* core genes (17) revealed GGRV to be its closest relative. An analysis of locally collinear blocks in Mauve (18) showed that BIV-ME 93/35, GGRV, and FV3 genomes are collinear. However, seven predicted FV3 ORFs are absent in BIV-ME 93/35 and GGRV, including FV3gorf13R, FV3gorf44R, FV3gorf49L, FV3gorf56R, FV3gorf65L, FV3gorf68L, and FV3gorf92R. The absence of these ORFs in BIV-ME 93/35 and GGRV explains their smaller reported genome sizes.

### Accession number(s).

The complete genome sequence of BIV-ME 93/35 has been deposited in GenBank under the accession no. KX185156.
